# Human TM9SF4 Is a New Gene Down-Regulated by Hypoxia and Involved in Cell Adhesion of Leukemic Cells

**DOI:** 10.1371/journal.pone.0126968

**Published:** 2015-05-11

**Authors:** Rosa Paolillo, Isabella Spinello, Maria Teresa Quaranta, Luca Pasquini, Elvira Pelosi, Francesco Lo Coco, Ugo Testa, Catherine Labbaye

**Affiliations:** 1 Department of Hematology, Oncology and Molecular Medicine of Istituto Superiore di Sanità, 00161, Rome, Italy; 2 Department of Biomedicine and Prevention, University of Rome “Tor Vergata”, Rome, Italy; 3 Fondazione Santa Lucia, Rome, Italy; Emory University, UNITED STATES

## Abstract

**Background:**

The transmembrane 9 superfamily protein member 4, TM9SF4, belongs to the TM9SF family of proteins highly conserved through evolution. TM9SF4 homologs, previously identified in many different species, were mainly involved in cellular adhesion, innate immunity and phagocytosis. In human, the function and biological significance of TM9SF4 are currently under investigation. However, TM9SF4 was found overexpressed in human metastatic melanoma and in a small subset of acute myeloid leukemia (AMLs) and myelodysplastic syndromes, consistent with an oncogenic function of this gene.

**Purpose and Results:**

In this study, we first analyzed the expression and regulation of TM9SF4 in normal and leukemic cells and identified TM9SF4 as a gene highly expressed in human quiescent CD34^+^ hematopoietic progenitor cells (HPCs), regulated during monocytic and granulocytic differentiation of HPCs, both lineages giving rise to mature myeloid cells involved in adhesion, phagocytosis and immunity. Then, we found that TM9SF4 is markedly overexpressed in leukemic cells and in AMLs, particularly in M2, M3 and M4 AMLs (i.e., in AMLs characterized by the presence of a more or less differentiated granulocytic progeny), as compared to normal CD34^+^ HPCs. Proliferation and differentiation of HPCs occurs in hypoxia, a physiological condition in bone marrow, but also a crucial component of cancer microenvironment. Here, we investigated the impact of hypoxia on TM9SF4 expression in leukemic cells and identified TM9SF4 as a direct target of HIF-1α, downregulated in these cells by hypoxia. Then, we found that the hypoxia-mediated downregulation of TM9SF4 expression is associated with a decrease of cell adhesion of leukemic cells to fibronectin, thus demonstrating that human TM9SF4 is a new molecule involved in leukemic cell adhesion.

**Conclusions:**

Altogether, our study reports for the first time the expression of TM9SF4 at the level of normal and leukemic hematopoietic cells and its marked expression at the level of AMLs displaying granulocytic differentiation.

## Introduction

The transmembrane 9 superfamily protein member 4 (TM9SF4) is one of the members of the TM9SF protein family characterized by a large N-terminal extracellular domain and nine-ten putative transmembrane domains, highly conserved through evolution [1–3]. Whether TM9SF proteins have been involved in cell adhesion, phagocytosis and autophagy in several species [3–10], little is known about the physiological role of the four TM9SF1-TM9SF4 proteins in mammals.

In human, TM9SF4 was first identified for its homology of sequence with *phg1A*, a Dictyostelium gene [4], recently found to control the expression and stabilization of the cell surface adhesion molecule SibA (Similar to Integrin Beta) which exhibits structural features present in mammalian integrin β (beta)-chains [10].

Then, TM9SF4 overexpression was observed in human malignant melanoma cells deriving from metastatic lesions and associated to the phenomenon of tumor cell cannibalism [11–13]. Cannibalism is an atypical phagocytic activity whose biological significance is still unknown [13], but that may be a crucial survival option for tumor in condition of starvation and hypoxia, both important components of cancer microenvironment [14, 15]. More recently, TM9SF4 was also described as a V-ATPase-associated protein in colon cancer cells [16].

In a study performed in a small subset of acute myeloid leukemia (AMLs) and myelodysplastic syndromes (MDS) characterized by the amplification of a chromosome 20 fragment (20q11.21) bearing among several genes the entire TM9SF4 gene, TM9SF4 mRNA was found overexpressed, thus suggesting a potential role for TM9SF4 in leukemogenesis [17].

In bone marrow (BM), oxygen supply is limited and hypoxia is considered as a physiological condition given the hierarchical organization of a small population of resting hematopoietic stem cells (HSCs) localized in hypoxic niches in BM microenvironment [18–21]. Leukemic cells can infiltrate the niches and use the homeostatic mechanisms of normal hematopoiesis leading to enhanced self-renewal and proliferation, quiescence, and resistance to chemotherapeutic agents [22–24]. In this context, adaptation of leukemic cells to the BM microenvironment is an important driving force in the clonal selection that leads to leukemic cells homing and mobilization, survival and relapse observed in leukemia, such as AMLs [22–26].

Cellular adaptation to hypoxia involves numerous mechanisms, including the transcriptional activation mediated by the hypoxia-inducible factors (HIFs), such as HIF-1α one of the most commonly expressed isoforms [27–30]. Hypoxia allows HIFs to escape from normoxia-mediated degradation in the cytoplasm and to translocate to the nucleus where they bind DNA sequences, called hypoxia-response elements (HREs), within the regulatory regions of target genes [31, 32].

Beside the importance of O_2_ supply, interactions of hematopoietic stem and progenitor cells (HSPCs) with other cells, growth factors and extracellular matrix (ECM) components of the BM microenvironment, also control homeostasis of HSPCs [33, 34]. In fact, the hematopoietic niches by producing a broad range of soluble factors and adhesion molecules can modulate HSPCs fate during normal hematopoiesis and BM regeneration [35, 36].

To investigate the role of human TM9SF4 in normal and leukemic hematopoietic cells, we analyzed the expression and regulation of TM9SF4 in hematopoietic progenitors cells (HPCs) first, and then in leukemic cell lines and primary leukemic cells obtained from AML patients.

In this study, we report for the first time the high expression of TM9SF4 in CD34^+^ HPCs and its regulation during monocytic (Mo) and granulocytic (G) proliferation and differentiation of these cells. A first screening for TM9SF4 gene expression performed in primary leukemic cells obtained from AML patients provides evidence that TM9SF4 is overexpressed in AMLs, in particular in those displaying granulocytic differentiation, such as M2, M3 and M4 subtypes of FAB classification, as compared to normal CD34^+^ HPCs.

By analyzing the impact of hypoxia on TM9SF4 expression, we then identified TM9SF4 as a new direct target gene of HIF-1α, downregulated in leukemic cells grown in hypoxia.

Furthermore, we showed that hypoxia-mediated downregulation of TM9SF4 in leukemic cells, as well as TM9SF4 knockdown mediated by RNA interference in these cells, is associated with a decrease of cell adhesion to fibronectin. Thus, our data provide the first evidence of a role for human TM9SF4 as a new hypoxia-responsive molecule involved in leukemic cell adhesion process, in line with TM9SF function observed in others species.

## Materials and Methods

### Ethics statement

The study was approved by the Ethics Committee of the Istituto Superiore di Sanità (National Institutes of Health in Rome, Italy) and the University Tor Vergata (Rome, Italy) (No. Prot. PRE 588/13; Prot. CE 393/13), and written informed consent was obtained from each patient prior to participation. The study was conducted in accordance with the Declaration of Helsinki.

### Cell cultures

From human cord blood (CB), CD34^+^ HPCs purification and unilineage monocytic (Mo) and granulocytic (G) liquid cultures in BIT 9500 serum-free medium (Stemcell Technologies Inc. Vancouver, BC, Canada) supplemented with (i) human low density lipoprotein (40μg/ml), FLT_3_ ligand (100 ng/ml), IL6 (10 ng/ml) and M-CSF (50 ng/ml) for Mo cultures; (ii) IL-3 (1 U/mL), GM-CSF (0.1 ng/mL), and G-CSF (500 U/mL) for G cultures, were performed as previously described [37, 38]. Erythroid (E) and megakaryocytic (Mk) proliferation and differentiation of CD34^+^ HPCs were performed as previously described [39, 40]. For morphologic analysis, HPCs were smeared on glass slides by cytospin centrifugation, stained with May-Grünwald-Giemsa and analyzed at 400X magnification under a microscope (Eclipse 1000, Nikon, Tokyo, Japan) equipped with a digital camera.

Human primary leukemic blasts obtained from AML patients, were maintained in culture in vitro in Iscove’s medium supplemented with 10% FCS, GM-CSF (10 ng/ml), SCF (50 ng/ml), IL-3 (10 ng/ml) (PeproTech Inc. Rocky Hill, NJ, USA). RNA and protein samples were previously prepared as described [41].

### Monocytic and granulocytic differentiation

Human myelomonocytic U937 cells were cultured with 1α-25-dihydroxyvitamin D3 (VIT.D3) (200 nM) for induction of monocytic differentiation/maturation, whereas promyelocytic HL-60 cells were cultured with all-trans retinoic acid (ATRA) (1 μM) (Sigma-Aldrich,St Louis, MO, USA) for induction of granulocytic differentiation/maturation.

Cell growth analysis, Mo and G membrane markers (CD11b, CD14 and CD54) expression by flow cytometry analysis were performed as described [42, 43].

### Hypoxia

To provide a mild (5% O_2_) or more severe (1% O_2_) hypoxic environment, cells were cultured and treated in sealed incubators calibrated for a constant hypoxic environment, 5% O_2_, 90% N_2_ and 5% CO_2_ referred to mild hypoxia, or to 1% O_2_, 94% N_2_ and 5% CO_2_ referred to severe hypoxia, at temperature 37°C. For physiological oxygenation or normoxia (N), cells were cultured in an incubator calibrated to 21% O_2_.

### Cell growth, apoptosis and cell cycle analysis

Cell proliferation, viability of the cells, apoptosis and cell cycle analysis were evaluated by using standard procedures and kit according manufacturer’s procedures (Annexin V-FITC apoptosis kit, 7-aminoactinomycin D and Cycletest Plus DNA detection kits, BD Pharmingen, San Jose, CA, USA).

### qRT-PCR analysis

Total RNAs were extracted using Trizol reagent and reverse transcribed by Moloney murine Leukemia virus reverse transcriptase (Invitrogen, Carlsbad, CA, USA) with random primers, as previously described [41]. TM9SF4 and HIF-1α mRNAs were detected by quantitative real-time PCR analysis (qRT-PCR) and normalized with the internal control β-Actin (ACTB), using commercial ready-to-use primers/probe mixes for TM9SF4 (Hs00207196_m1), HIF-1α (Hs00153153_m1) and ACTB (20X, 4310881E) (Applied Biosystems, Foster City, CA, USA) according to the manufacturer’s procedure and TaqMan technology [41]. Duplicate assays were performed with RNA samples obtained from at least three independent experiments.

### Flow cytometry analysis

To evaluate TM9SF4 protein expression, cells were fixed and permeabilized with Fix and Perm reagents (Thermo Fisher Scientific, Austria) before incubation with either TM9SF4 polyclonal antibody (pAb) (1:25), a purified pAb raised against a peptide mapping within an internal region of human TM9SF4 (S-20, Santa Cruz, CA, USA), then incubated with a secondary antibody Alexa-Fluor 647 (1:1000) (Life technologies, Italy), or with AlexaFluor-647 used alone as a negative control. Fluorescence emission was analyzed using the FACSCanto flow cytometer (Becton Dickinson, Franklin Lakes, NJ, USA) and the DIVA software for acquisition and analysis (Becton Dickinson). The mean fluorescence intensity (MFI) indicates the ratio between the geometric mean fluorescence values observed in labeled cells and cells labeled with a negative control, gated on FSC/SSC dot plot to select viable cells.

### Western blot analysis

Western blot analysis were performed by using (i) 40 μg of total protein extracts loaded on 10% polyacrylamide gels for TM9SF4, or (ii) 30 μg of nuclear protein extracts loaded on 7.5% polyacrylamide gels for HIF-1α, then transferred on membrane by using Transblot-Turbo transfer system, according manufacturer’s procedures (Bio-Rad, Hercules, CA, USA). Anti-TM9SF4 pAb (ab98879, Abcam, Cambridge, UK) and anti-HIF1α pAb (AF1935, R&D Systems, Minneapolis, MN, USA) were used according standard methods. Monoclonal antibodies anti-actin (Sigma-Aldrich, St Louis, MO, USA) and anti-nucleolin (Oncogene Research Products, Boston, MA, USA) were respectively used as internal control of total and nuclear proteins expression.

### Immunofluorescence analysis

5x10^4^ cells were fixed in formaldehyde 3.7% and permeabilized with 0.5% PBS-triton X-100 then plated by cytospin (Cytospin3, SHANDON) on polylysine-coated microscope slides. After blocking with PBS-triton 0.1%-goat serum (Biorad) 3% (15 minutes, room temperature), cells were stained over-night at +4°C with TM9SF4 primary antibody (1:50) (S-20, Santa Cruz). Subsequently, slides were washed 3–4 times with PBS, incubated with AlexaFluor-647 (1:1000) (donkey anti–goat IgG, Invitrogen) for 1 hour at room temperature, washed again and stained with 4’,6-Diamidino-2-Phenylindole (DAPI) to label the nuclei (10 minutes, room temperature). Fluorescence was detected using an Olympus FV-1000 spectral confocal microscope.

### Chromatin immunoprecipitation (ChIP) assays

2x10^6^ cells were crosslinked to DNA and sonicated before using the ChIP assay kit (Upstate, Charlottesville, VA, USA) according to the manufacturer’s procedure, and protein-DNA complexes were immunoprecipitated with the anti-HIF1α-ChIPgrade polyclonal antibodies (pAbs) (ab2185, Abcam, Cambridge, UK) or with the unrelated anti-c-abl monoclonal antibody (mAb) (Oncogene Research Products, Boston, MA, USA) used as a negative control, or protein-A sepharose only. A genomic region of 163 bp containing the HRE sequence *ACGTG* [31, 32] in the putative TM9SF4 promoter region [NM_014742] was amplified in the immunoprecipitates by PCR using specific primers flanking the HRE site in the Prom-TM9SF4 region (forward, from -153 of the start codon ATG: 5’-CAGACTGTCGAGCAGGAG-3’; and reverse to -7: 5’-GCCGTCGCCATCTTGGAT-3’) and PCR conditions: 94°C/30s; 40 cycles of (95°C/30s; 58°C/30s; 72°C/35s); 72°C/1 min. PCR products were loaded on 1% agarose-TBE(1X) gel and bands were visualized by using ethidium bromure coloration. In the immunoprecipitates no relevant DNA sequences were detected by PCR amplification of a 172 bp genomic sequence without any HRE site and localized upstream to the Prom-TM9SF4 region, by using primers: forward at -562: 5’-(TCACAGATGGGAATGAGG)-3’and reverse at -390: 5’-(AGCAGTACGACTCCAAGA)- 3’ and PCR conditions 40 cycles of (95°C/30s; 54°C/30s; 72°C/35s); 72°C/1 min. Non relevant cellular DNA sequences were detected by amplification of a GAPDH coding region using primers and PCR conditions as described [44].

### Promoter assays

TM9SF4 promoter activity was evaluated by luciferase assays. A 235 bp DNA fragment of the putative promoter of TM9SF4 (NM_014742) was PCR-amplified from genomic DNA using the primers forward 5’-AGTTTCTGCCAGGAGCTAAT-3’ and reverse

5’-CTTGGATCCACGTGTCGC-3’, and cloned upstream to the luciferase gene into pGL3Basic (pGL3Basic/Prom-TM9SF4) and pGL3Promoter (pGL3Prom/Prom-TM9SF4) vectors (Promega, Madison, WI, USA). By mutagenesis of the HRE site into the pGL3Prom/Prom-TM9SF4 vector using, according manufacturer’s instructions, the QuickChange Site-Directed mutagenesis kit (Stratagene, La Jolla, CA, USA), we prepared the HRE mutated Prom TM9SF4 vector (pGL3Prom/Mut-Prom-TM9SF4). Human HIF-1α full length cDNA was cloned into a pcDNA3.1(+) expression vector (pcDNA3.1/HIF-1α vector from GenScript, Piscataway, NJ, USA). All vectors were checked by automated sequencing.

In luciferase assay experiments, 293T cells were transfected using Lipofectamine 3000 (Life Technology, Italy), with a Renilla luciferase vector (50 ng), together with luciferase vectors described above (100 ng) and, where indicated, with the pcDNA3.1/HIF-1α vector. Luciferase activity was measured 48 hrs post-transfection with the Dual Luciferase Reporter System (Promega, Madison, WI, USA) according to the manufacturer’s instructions, by using Microlite TLX1 (Dynatech Laboratoires, Chantilly, CA) and then normalized for Renilla Luciferase activity. Data are presented as mean values obtained from at least three independent experiments.

### Human TM9SF4 full length cDNA cloning

Human TM9SF4 full length cDNA was first cloned and sequenced into a pcDNA3.1(+) expression vector (pcDNA3.1/TM9SF4 vector from GenScript, Piscataway, NJ, USA). Then, we performed transfection of 293T cells with pcDNA3.1/TM9SF4 vector by using lipofectamine method as described in section above, to prepare total protein extracts from 293T(pcDNA3.1/TM9SF4) expressing cells to use as a positive control (+) of TM9SF4 protein expression in western blot analysis.

### Dimethyloxalyl Glycine treatment of leukemic cells

Dimethyloxalyl Glycine (DMOG) treatment of U937 or HL-60 cells (25x10^4^ cells/ml) was performed in normoxia by using 200 μM of DMOG (R&D Systems, Minneapolis, MN, USA) for 48 hrs, as compared to HIF-1α expressing- U937 and HL-60 cells cultured in hypoxia (Hx, 1% O_2_), as described [45].

### Knockdown of TM9SF4 and HIF-1α expression by RNA interference

TM9SF4 gene was silenced with synthesized small interfering ribonucleic acids (siRNA) TM9SF4-siRNA (Sil. Sel. siRNA TM9SF4 human, INV, STD, 5NM, cod. 4392420 from Life Technologies, Italy). 1,5x10^6^ U937 cells were transfected with 100 nM of TM9SF4-siRNA, or non-targeting control siRNA (c-siRNA) (Sil. Sel. siRNA Negative Control, 1.5 NM, cod. 4390843, from Life Technologies, Italy) by using Lipofectamine 3000 according to the manufacturer’s protocol (Life Technologies, Italy). U937(TM9SF4-siRNA)- and U937(c-siRNA)- transfected cells were maintained 48 hrs in culture under normoxia; TM9SF4 downmodulation was controlled at protein level by Western blot analysis.

HIF-1α gene was silenced with synthesized HIF-1α-siRNA (ON-TARGET plus SMART pool siR J004018-10 HIF-1α, from Dharmacon, CO, USA). U937 cells were transfected twice with 100 nM of HIF-1α-siRNA, or non-targeting control siRNA (c-siRNA) (ON-TARGET plus Non-Targeting Pool D-001810-10-05, from Dharmacon, USA) by using Lipofectamine 3000. Following the second transfection, U937(HIF-1α-siRNA)- and U937(c-siRNA)- transfected cells were maintained 24 hrs in culture under hypoxia (1% O_2_), as described [46]. HIF-1α downmodulation was controlled by Western blot analysis of nuclear extracts prepared from U937(HIF-1α-siRNA) cells, as compared to U937(c-siRNA) control cells.

### Cell adhesion assays

We performed adhesion assays by using fibronectin (Fn)-coated-24 wells-plates (BD BioCoat Cellware). Cells (1x10^3^ cells per well) were seeded in cultures 48 hrs in hypoxia, as compared to normoxia. Wells were then gently washed twice with PBS to remove non adherent cells and cell adhesion was measured by counting the remaining adherent cells. Pictures for each condition were taken with a digital camera (C-4040zoom 4.1 megapixel, Olympus) linked to the optical microscope used to count cells (IX51, Olympus).

Adhesion assays were also performed with U937(TM9SF4-siRNA) cells previously prepared by transfection of U937 cells with siRNA-TM9SF4, as compared to siRNA-control U937(c-siRNA) cells, and seeded under normoxic conditions on Fn-coated plates as described above.

### Statistical analysis

Results are presented as mean ± SEM of three independent experiments. Student’s t-test was used to calculate the statistical significance (p-value of more than 0.05 was considered not statistically significant).

## Results

### TM9SF4 is expressed in CD34^+^ HPCs and during monocytic (Mo) and granulocytic (G) differentiation of HPCs, but is overexpressed in AMLs

**Fig 1:**
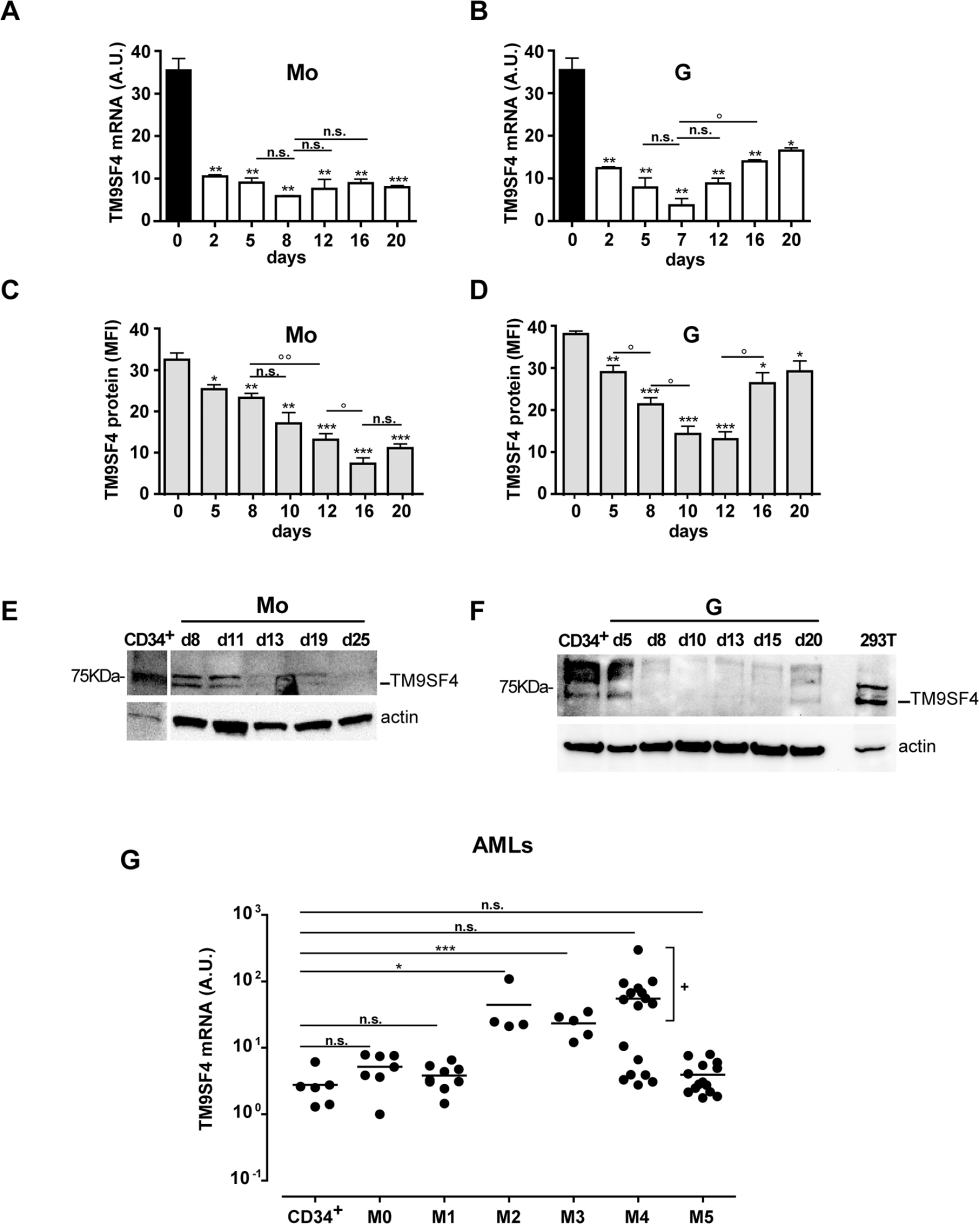
TM9SF4 is expressed during Mo and G proliferation and differentiation of HPCs, but is overexpressed in AMLs. (A, B) Real time PCR analysis of TM9SF4 mRNA expression during selective Mo and G proliferation and differentiation of CD34^+^ HPCs (day 0). (C-F) Analysis of TM9SF4 protein expression is shown by flow cytometry (C, D) and Western blot (E, F) analysis in Mo and G differentiating HPCs. (G) Real time PCR analysis of TM9SF4 mRNA expression in primary leukemic cells of AMLs pertaining from M0 to M5 subtypes of FAB classification, as compared to normal CD34^+^ HPCs. (A—D) The results of three independent experiments (mean ± SEM values) are shown; n.s. is for not significant; significance *, **, *** are p<0.05, p<0.01, p<0.001 respectively and as compared to day 0; °, °° are p<0.05, p<0.01 respectively and between indicated days. (A, B, G) A.U. is for arbitrary units. (C, D) MFI is for Mean Fluorescence Intensity. (E, F) One representative experiment out of three is shown; actin is an internal control. (F) 293T is for protein extract prepared from 293T(pcDNA3.1/TM9SF4) overexpressing cells used as a positive control of TM9SF4 protein expression. (G) Results are presented as scatter plot. Mean is indicated; significance *, ^+^ and *** are p<0.05 and p<0.001 respectively.

We first analyzed TM9SF4 mRNA and protein expression in human cord blood CD34^+^ HPCs grown in culture conditions allowing their selective unilineage erythroid (E), megakaryocytic (Mk), monocytic (Mo) or granulocytic (G) differentiation and maturation that we performed under mild hypoxia (5% O_2_), a condition that mimics the physiological oxygen tension observed during HPCs differentiation, as previously described [37–40]. Three independent experiments were performed for each lineage and RNA samples were generated at sequential days from HPCs differentiating and maturing through the E, MK, G and Mo pathways. By using real-time PCR analysis, we found that TM9SF4 mRNA expression is high in CD34^+^ HPCs (day 0), then significantly decreases (p< 0.01, **) during the first days (day 0–2) of Mo and G proliferation and differentiation (Fig 1A and 1B). Later, whereas we cannot detect any significant modulation of TM9SF4 mRNA expression levels from day 5 to day 20 during Mo differentiation and from day 5 to day 12 during G differentiation (Fig 1A and 1B; p is not significant), TM9SF4 mRNA expression significantly increases during G maturation (day 12 to 20, Fig 1B; p < 0.05, °).

Instead, TM9SF4 mRNA expression is almost undetectable through E and Mk proliferation and differentiation (data not shown).

Flow cytometry and Western blot analysis showed that, as observed for TM9SF4 mRNA, higher TM9SF4 protein level is found in quiescent CD34^+^ HPCs than in differentiated Mo or G cell progeny (day 0 and CD34^+^, Fig 1C–1F). TM9SF4 protein levels that significantly decrease during Mo (from day 0 to 20) and G (from day 0 to 12) differentiation pathways (Fig 1C and 1D), significantly increase at the later, terminal stages of G differentiation (day 12–20, Fig 1D). This pattern of TM9SF4 protein expression in Mo and G differentiating HPCs was also confirmed by Western blot analysis (Fig 1E and 1F).

These findings identify TM9SF4 as a new molecule expressed in quiescent human CD34^+^ HPCs and regulated in a lineage-specific way, during Mo and G differentiation.

Then, we performed a first screening for TM9SF4 mRNA expression in primary leukemic blasts obtained from AML patients pertaining from the M0 to M5 subtypes following the FAB classification (Fig 1G). Our data show that TM9SF4 mRNA is significantly overexpressed in AMLs pertaining to the M2, M3 subtypes and, for some AMLs, to the M4 subtype, as compared to CD34^+^ HPCs (Fig 1G). It is of interest to note that TM9SF4 mRNA expression in M4 AMLs is highly heterogeneous, with evidence of two groups, one “low” exhibiting a no significant overexpression and the other “high” showing a significant overexpression (Fig 1G, p< 0.05, **+**) compared to normal CD34^+^ cells (Fig 1G).

We also analyzed TM9SF4 expression during Mo and G differentiation of leukemic cell lines U937 and HL-60 that growing under normoxic condition, are respectively used as models of vitamin D3 (VIT.D3)-inducible Mo differentiation and all trans retinoic acid (ATRA)-inducible G differentiation, as previously described [42, 43].

**Fig 2:**
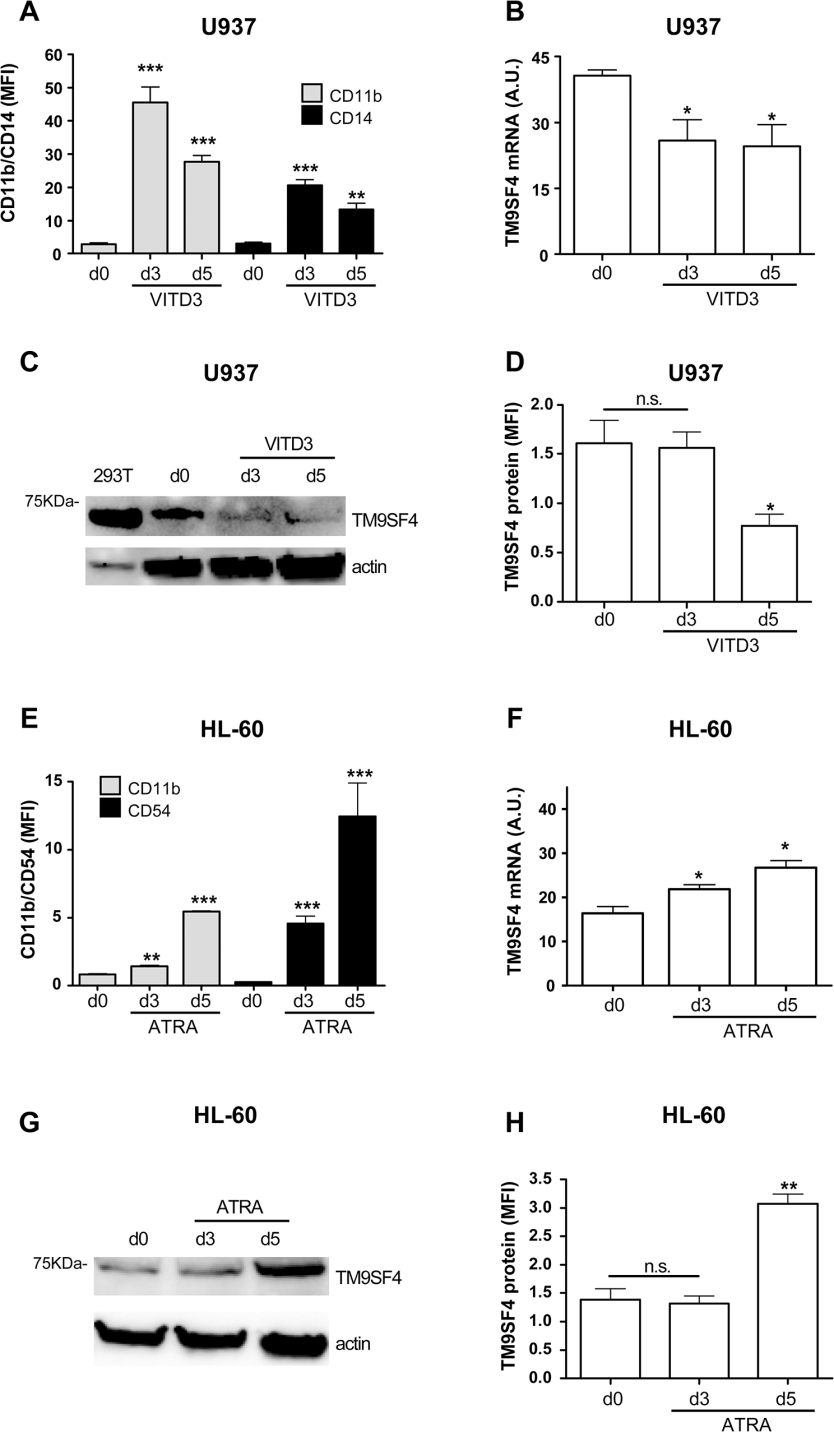
TM9SF4 decreases during vitamin D3-induced Mo differentiation of U937 cells, but increases during ATRA-induced G differentiation of HL-60 cells. (A) Flow cytometry analysis of specific Mo markers CD11b and CD14 expression was performed from day 0 to 5 (d5) in U937 cells treated with vitamin D3 (VITD3) to induce Mo differentiation, as compared to untreated U937 control cells (d0). (B) Real time PCR analysis of TM9SF4 mRNA expression during VITD3- induced Mo differentiation of U937 cells. (C, D) Western blot (C) and flow cytometry analysis (D) of TM9SF4 protein expression in VITD3-treated U937 cells, as compared to untreated control U937 cells (d0). (E) Flow cytometry analysis of G markers CD11b and CD54 was performed from day 0 to 5 (d5) in HL-60 treated with all trans retinoic acid (ATRA) for G differentiation, as compared to untreated HL-60 control cells (d0). (F) Real time PCR analysis of TM9SF4 mRNA expression during ATRA- induced G differentiation of HL-60 cells. (G, H) Western blot (G) and flow cytometry analysis (H) of TM9SF4 protein expression in ATRA-treated HL-60 cells, as compared to untreated control HL-60 cells (d0). (A, B, D, E, F, H) The results of three independent experiments (mean ±SEM values) are shown; *, **, *** are p<0.05, p<0.01, p<0.001 respectively; n.s. is for not significant. (A, D, E, H) MFI is for Mean Fluorescence intensity. (B, F) A.U. is for arbitrary units. (C, G) One representative experiment out of three is shown; actin is shown as an internal control; (C) 293T is for 293T(pcDNA3.1/TM9SF4) overexpressing cells used as a positive control of TM9SF4 protein expression.

In U937 cells that undergo VIT.D3-induced Mo differentiation, as shown by the increase expression of Mo markers CD11b and CD14 (Fig 2A), TM9SF4 mRNA and protein expression decreases compared to untreated (d0) cells (Fig 2B–2D). During ATRA-induced G differentiation of HL-60 cells that we controlled by analyzing G marker expression CD11b and CD54 (Fig 2E), TM9SF4 mRNA and protein expression increases compared to untreated (d0) cells (Fig 2F–2H). These data mimic those observed during Mo and terminal G differentiation of normal HPCs (Fig 1).

Altogether, our data report that TM9SF4 is a new gene expressed in CD34^+^ HPCs, differentially expressed during Mo and G differentiation, and overexpressed in some AMLs.

### Hypoxia downmodulates TM9SF4 expression in leukemic cells, as HIF-1α is a repressor of TM9SF4 in these cells.

**Fig 3:**
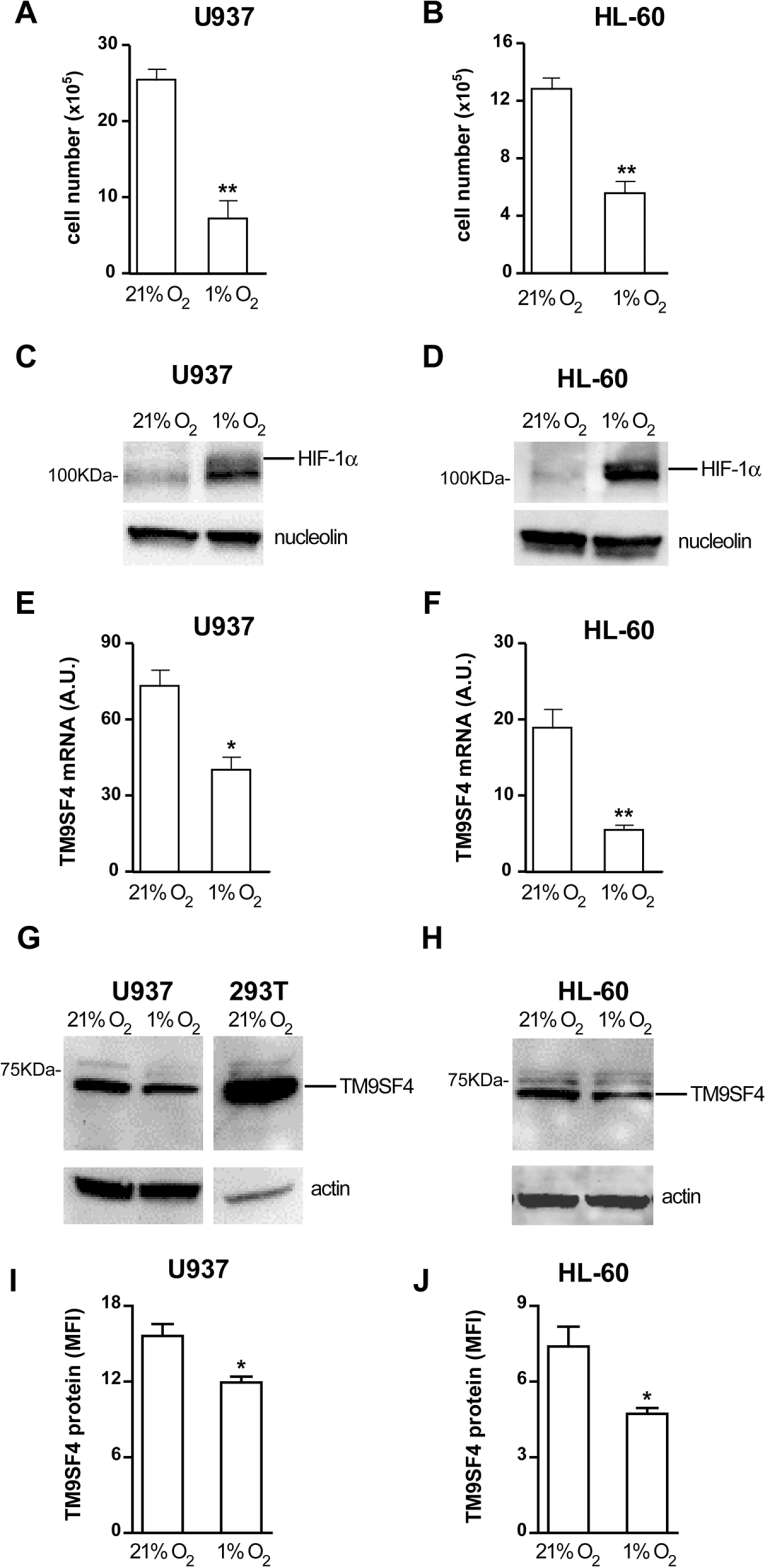
Hypoxia that activates HIF-1α, downmodulates TM9SF4 expression in leukemic cells. (A, B) Cell growth analysis of U937 (A) and HL-60 (B) cells grown 48 hours in hypoxia (1% O_2_), as compared to normoxia (21% O_2_). (C, D) Western blot analysis of HIF-1α nuclear protein expression in U937 (C) and HL-60 (D) cells cultured in hypoxia (1% O_2_), as compared to normoxia (21% O_2_). (E, F) Real time PCR analysis of TM9SF4 mRNA expression in U937 (E) and HL-60 (F) cells in hypoxia, as compared to normoxia. (G—J) TM9SF4 protein expression levels were analyzed by Western blot (G, H) and flow cytometry (I, J) analysis in U937 (G, I) and HL-60 (H, J) cells in hypoxia, as compared to normoxia. (A, B, E, F, I, J) The results of three independent experiments (mean ± SEM values) are shown; *, ** are p<0.05, p<0.01 respectively. (C, D, G, H) One representative experiment out of three is shown; nucleolin is an internal control of nuclear protein extracts (C, D); actin is an internal control of total protein extracts (G, H); 293T is for 293T(pcDNA3.1/TM9SF4) overexpressing cells, used as a positive control of TM9SF4 protein expression (G). (E, F) A.U. is for arbitrary units.

We investigated the impact of hypoxia on TM9SF4 expression in U937 and HL-60 leukemic cells grown for 48 hrs in condition of hypoxia (1% O_2_), as compared to normoxia (21% O_2_). First, we analyzed the cell growth rate and apoptosis of U937 and HL-60 cells maintained in hypoxia several days, as compared to normoxia. Whereas a significant decrease of cell growth was observed in both cell lines in hypoxia (Fig 3A and 3B), we did not detect any significant apoptosis of U937 and HL-60 cells up to 48 hours of culture in hypoxia, as compared to normoxia (data not shown), and then we examined HIF-1α activation and TM9SF4 expression in these cells.

**Fig 4:**
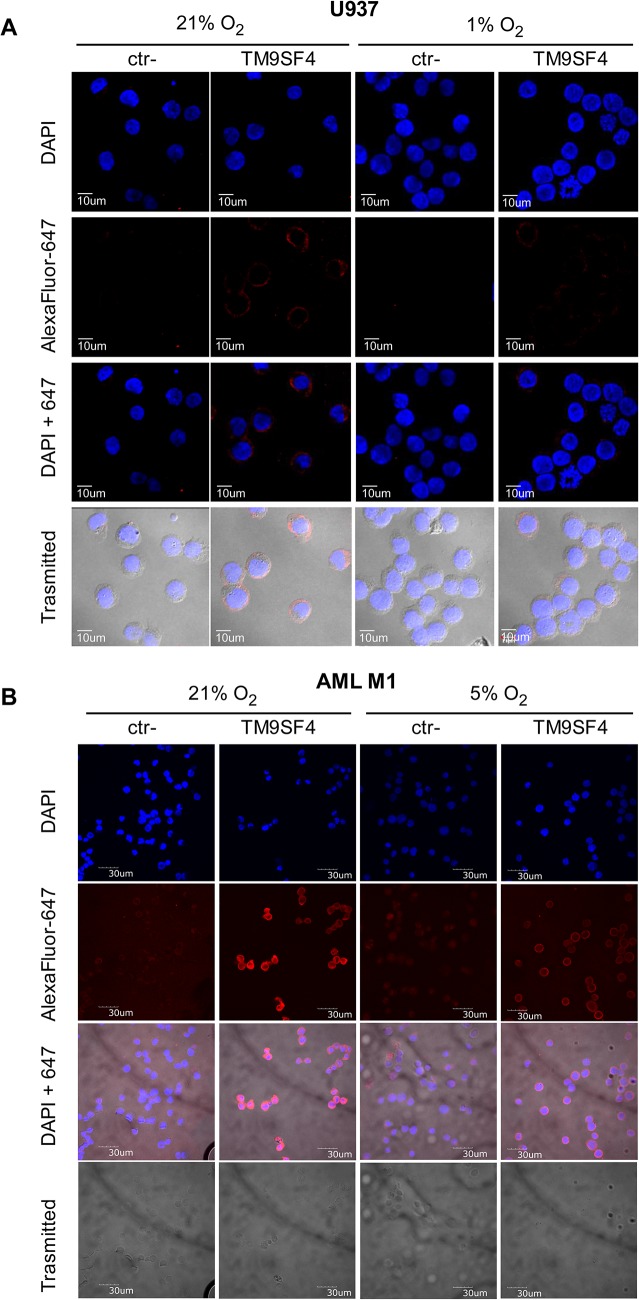
Hypoxia downmodulates TM9SF4 expression in leukemic cells. (A, B) The decrease of TM9SF4 protein expression in U937 cells (A) and in primary leukemic cells from AML M1 (B) cultured under hypoxic (1% and 5% O_2_, respectively) compared to normoxic (21% O_2_) conditions is shown by immunofluorescence analysis of TM9SF4. ctr- is for no primary antibody negative control. DAPI shows nuclear staining; AlexaFluor-647 shows TM9SF4 protein staining; DAPI+647 is the merge of AlexaFluor-647 and DAPI; Trasmitted shows the phase-contrast microscopy fields; Scale bars indicated are 10μm (A) and 30μm (B).

Western blot analysis showed HIF-1α nuclear protein accumulation in both U937 and HL-60 cells in hypoxia, as compared to normoxia (Fig 3C and 3D). TM9SF4 expression is downregulated in leukemic cells grown in hypoxia, at both mRNA and protein levels, as shown respectively by real time PCR analysis (Fig 3E and 3F) and Western blotting (Fig 3G and 3H), flow cytometry (Fig 3I and 3J) or immunofluorescence analysis (Fig 4A). The decrease of TM9SF4 expression was more pronounced in HL-60 than in U937 cells.

However, it is of interest to note that in normoxia TM9SF4 mRNA expression level is higher in Mo leukemic U937 cells, as compared to HL-60 leukemic cells (Fig 3E and 3F, panels 21% O_2_).

Furthermore, the hypoxia-mediated downregulation of TM9SF4 found in leukemic cell lines was also observed by immunofluorescence analysis in primary leukemic cells maintained 16 hrs in condition of mild hypoxia (5% O_2_), as compared to normoxia (Fig 4B).

Because most of target genes of HIFs are transcriptionally activated in hypoxia, we further investigated the possible interaction between HIF-1α and the promoter region of the TM9SF4 gene.

**Fig 5:**
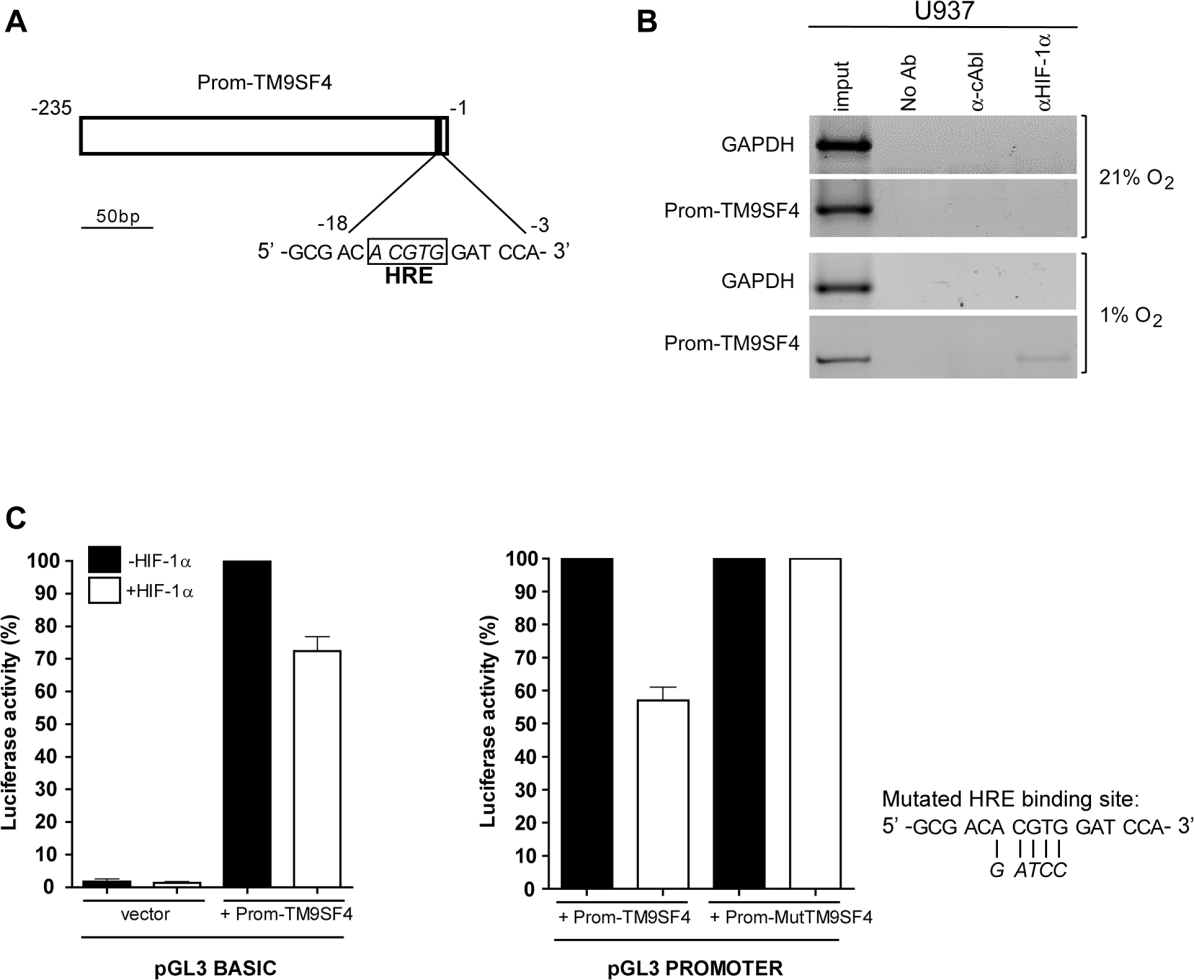
In hypoxia, TM9SF4 is a direct target gene of HIF-1α in leukemic cells. (A) HIF-1α DNA binding site (HRE) identified on TM9SF4 partial promoter sequence is shown. (B) Chromatin immunoprecipitation (ChIP) experiments were performed by using protein extracts of U937 cells cultured in hypoxia (panels 1% O_2_), as compared to normoxia (panels 21% O_2_), immunoprecipitated with the anti(α)-HIF-1α or with an unrelated α-cAbl antibodies, or no antibody (No Ab), and analyzed by PCR amplification of the TM9SF4 region surrounding the HRE site (Prom-TM9SF4) or a GAPDH coding region used as an internal control. Input: PCR on 0.02% of total chromatin. One representative experiment of three is shown. (C) Promoter activity assays were performed first (*left panels*) by using TM9SF4 upstream DNA sequence subcloned in the pGL3Basic promoterless luciferase vector (Prom-TM9SF4) and transfected into 293T cells in the presence (white bars) or absence (black) of a HIF-1α expression vector, as compared to empty pGL3Basic vector (vector); then (*right panels*) by using Prom-TM9SF4, or the same region with a mutated HRE binding site (Prom- MutTM9SF4), cloned in the pGL3Promoter reporter vector containing a minimal promoter, and transfected with or without a HIF-1α expression vector. Data of luciferase activity detected are mean ± S.E.M. values of 3 independents experiments.

First, we analyzed the DNA region, putative partial promoter region of TM9SF4 (Prom-TM9SF4) (NM_014742) and found the sequence 5´- ACGTG -3´ between positions -18 and -3 of this region (Fig 5A), which is related to the previously described HIF-1α DNA-binding consensus sequence or Hypoxia Response Element (HRE) [31, 32].

Then, we evaluated whether HIF-1α was recruited at the Prom-TM9SF4 region by chromatin immunoprecipitation (ChIP) experiments performed by using nuclear extracts of U937 cells in hypoxia and expressing HIF-1α, as compared to U937 cells in normoxia not expressing HIF-1α (Fig 5B).

Protein-DNA complexes were immunoprecipitated with the anti-HIF-1α monoclonal ChIP-grade antibody (α-HIF-1α), no antibody (No Ab) or the unrelated anti-c-abl monoclonal antibody (α-cAbl) used as a negative control (Fig 5B). The immunoprecipitates were amplified by PCR using primers flanking the putative HRE in the partial promoter region of TM9SF4 (Fig 5A, Prom-TM9SF4), or primers recognizing a region upstream of this sequence, which does not contain any identifiable HRE site (data not shown).

Our ChIP assays indicated that HIF-1α is associated with the region surrounding the putative HRE binding site into the Prom-TM9SF4 genomic region in U937 cells in hypoxia (Fig 5B, panels 1% O_2_), but not in U937 cells in normoxia (Fig 5B, panels 21% O_2_).

To assess the ability of the Prom TM9SF4 genomic region to promote transcription, we cloned this region containing the HRE site (Prom-TM9SF4) or a mutant version (Prom-MutTM9SF4) into reporter vectors such as pGL3Basic vector first, then into a reporter vector containing an artificial minimal promoter, pGL3Promoter, and performed luciferase assays in presence or not of pcDNA3.1/HIF-1α expressing vector (Fig 5C). Our data show that HIF-1α repressed luciferase activity of the Prom-TM9SF4 vector, but not that of the Prom-MutTM9SF4 vector (Fig 5C).

The luciferase activity detected for the pGL3basic-Prom-TM9SF4 construct, inhibited of about 30% by HIF-1α (Fig 5C, left panel), identifies the DNA sequence cloned here as a promoter region of the gene TM9SF4. Furthermore, by using the pGL3Prom-Prom-TM9SF4 construct, we observed about 40% of inhibition of the luciferase activity of Prom-TM9SF4 in presence of HIF-1α, whereas we cannot detect any inhibition of the luciferase activity of the construct pGL3Prom-Prom-MutTM9SF4 construct in presence of HIF-1α (Fig 5C, right panel).

Altogether, we found a HRE DNA-binding site in the newly identified partial promoter region of TM9SF4, Prom-TM9SF4, and showed that HIF-1α is a direct transcriptional repressor of TM9SF4 mRNA expression in leukemic cells cultured in hypoxia, as compared to normoxia.

### DMOG stabilization of HIFs-α and HIF-1α mRNA silencing demonstrate the direct hypoxia-mediated repression of TM9SF4 in leukemic cells

We used a prolyl hydroxylase inhibitor such as DMOG (dimethyloxalyl Glycine) [45] to pharmacologically stabilize HIFs-α proteins in leukemic cells grown under normoxia and analyzed the effects on TM9SF4 expression levels.

**Fig 6:**
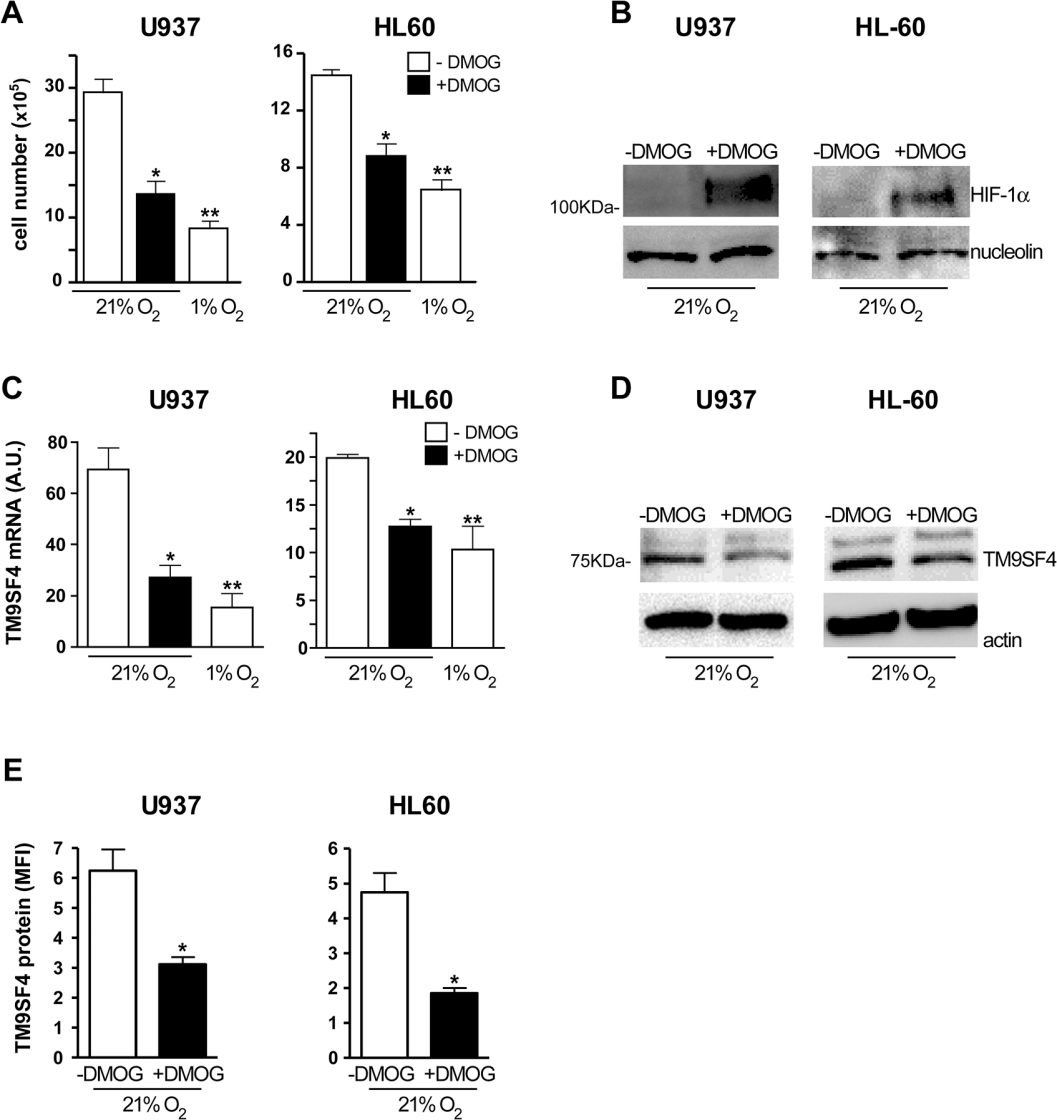
Stabilization of HIF-1α by DMOG treatment of leukemic cells grown in normoxia, mimics hypoxia and then decreases TM9SF4 expression. (A) DMOG treatment of U937 and HL-60 cells (+DMOG) compared to untreated cells (-DMOG) in normoxia (21% O_2_), decreases leukemic cell growth, thus mimicking hypoxia (1% O_2_). (B) DMOG treatment of U937 and HL-60 cells cultured in normoxia stabilizes HIF-1α nuclear protein expression, as shown by western blot analysis. (C-E) DMOG treatment of U937 and HL-60 cells (+DMOG) compared to untreated cells (-DMOG) in normoxia (21% O_2_), decreases TM9SF4 expression at both mRNA level, as shown by real time PCR analysis (C), and protein level analyzed by Western blotting (D) or flow cytometry analysis (E), as found in hypoxia (1% O_2_). (A, C, E) The results of three independent experiments (mean + SEM values) are shown; *, ** are p<0.05, p<0.01 respectively. (B) Nucleolin is used as an internal control of nuclear protein extracts; one representative experiment out of three is shown. (C) A.U. is for arbitrary units. (D) Actin is shown as internal control of total protein extracts; one representative experiment out of three is shown.

In normoxia, DMOG treatment of U937 and HL-60 cells induces a decrease of cell growth (Fig 6A) and HIF-1α nuclear proteins accumulation (Fig 6B), as observed in hypoxia (1% O_2_) (Fig 3C and 3D). DMOG decreases TM9SF4 expression at mRNA (Fig 6C) and protein (Fig 6D and 6E) level in both U937 and HL-60 cells, as previously found in hypoxia (Fig 3E–3J). Thus, DMOG treatment of leukemic cells in normoxia mimics the effects of hypoxia on these cells and confirms the hypoxia-mediated regulation of TM9SF4.

**Fig 7:**
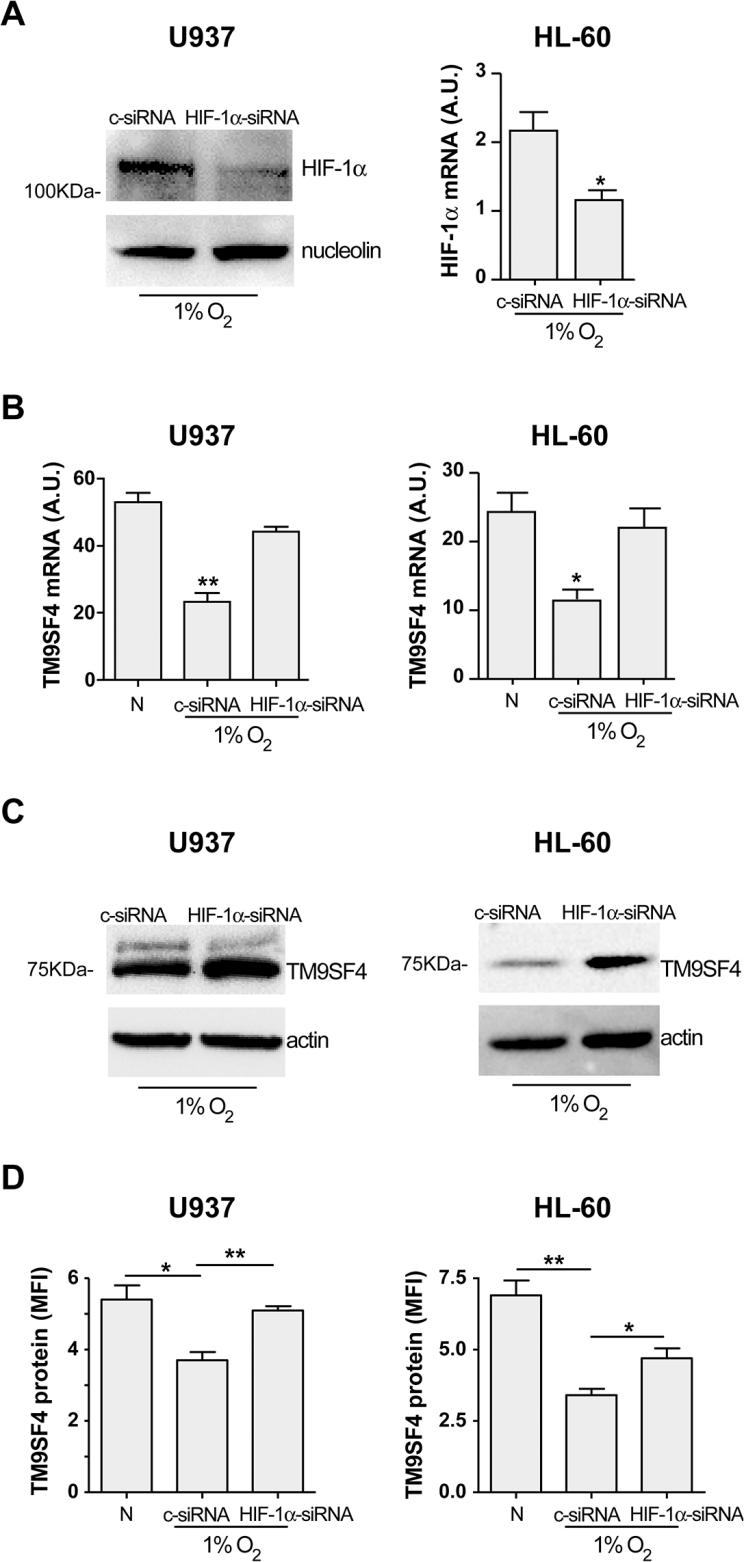
In hypoxia, HIF-1α is a direct transcriptional repressor of TM9SF4 in leukemic cells. (A) Performed under hypoxic condition (1% O_2_), knockdown of HIF-1α in U937 and HL-60 cells by using HIF-1α small interfering RNAs (HIF-1α-siRNA), as compared to control siRNA (c-siRNA), was controlled at protein level by Western blot analysis in U937 cells (left panel) and at mRNA level by real time PCR analysis in HL-60 cells (right panel). (B-D) TM9SF4 expression in U937(HIF-1α-siRNA) and HL-60(HIF-1α-siRNA) cells, as compared to U937(c-siRNA) and HL-60(c-siRNA) control cells, cultured in hypoxia (1% O_2_) compared to normoxia (N), was analyzed at mRNA level by real time PCR analysis (B) and at protein level by western blot (C) and flow cytometry (D) analysis in U937 and HL-60 cells. (A HL-60, B, D) The results of three independent experiments (mean ± SEM values) are shown; *, ** are p<0.05, p<0.01 respectively. (A U937, C) One representative experiment out of three is shown; nucleolin is used as an internal control of U937 nuclear protein extracts; actin is shown as internal control of total protein extracts (C).

To confirm the specific binding of HIF-1α on its target gene TM9SF4 in U937 and HL-60 leukemic cells grown in hypoxia, we silenced HIF-1α expression in these cells. Specific silencing of HIF-1α mRNA, controlled by the decrease of HIF-1α protein level in HIF-1α-siRNA transfected-U937 cells (Fig 7A, U937) and by the decrease of HIF-1α mRNA expression in HL-60 cells (Fig 7A, HL-60), increases TM9SF4 mRNA (Fig 7B) and protein (Fig 7C and 7D) expression in these cells, as compared to c-siRNA transfected control cells.

TM9SF4 is a new direct specific target gene of HIF-1α, downregulated in leukemic cells in hypoxia.

Then, we investigated whether hypoxia may have an impact on the modulation of TM9SF4 expression observed during VIT.D3-induced Mo differentiation of U937 cells or ATRA-induced G differentiation of HL-60 cells (Fig 2). U937 and HL-60 cells were grown in hypoxia (1% O_2_) and induced to differentiation with VIT.D3 or ATRA respectively, under hypoxic condition (S1 Fig).

Hypoxia stimulates VIT.D3-induced differentiation of U937 cells or ATRA-induced differentiation of HL-60 cells, as shown by the increased expression of Mo (CD11b) or G (CD54) markers for, respectively, U937 (S1A Fig) and HL-60 (S1B Fig) cells at levels higher than in normoxia (Fig 2A and 2E). Under hypoxia, HIF-1α nuclear protein level that accumulates in both U937 and HL-60 cell lines (day 0) and during their differentiation up to day 5 (S1C and S1D Fig), decreases TM9SF4 protein expression level in the U937 and HL-60 cells (day 0, S1E and S1F Fig), as compared to normoxic cells (Fig 3G–3J). Then, we only detect a low level of TM9SF4 expression either by western blot (S1E Fig) or flow cytometry analysis (not shown) during VIT.D3-induced differentiation of U937 cells performed in hypoxia. TM9SF4 expression that is downregulated in HL-60 cells in hypoxia, as compared to normoxia (Fig 3F and 3H and 3J), is always expressed in these cells, but also in HIF-1α-siRNA transfected-HL-60 cells, during their ATRA-induced differentiation performed in hypoxia (S1F and S1G Fig).

Altogether, our data indicate two main mechanisms of regulation of TM9SF4 expression in leukemic cells, one related to the cell differentiation and the other one related to hypoxia.

### TM9SF4 downregulation is associated with a loss of cell-adhesion of leukemic cells

TM9SF proteins in different animal species have been shown to be mainly involved in cell adhesion, but in human a role for TM9SF4 in this process has never been reported. Here, we investigated a putative role of TM9SF4 in the process of cell adhesion in human Mo leukemic cells.

First, we performed cell adhesion assays on U937 cells by using plates coated with different substrates, such as laminin, fibronectin, gelatin and stromal cells (e.g. MS5 cells), and found that U937 cells had a higher affinity for adhesion to fibronectin (Fn) than to other substrates (data not shown).

Then, we plated U937 cells on Fn-coated wells and performed cultures in hypoxia (1% O_2_), as compared to normoxia (21% O_2_), to count the number of adherent leukemic cells found per well after 48 hrs of culture in hypoxia, as compared to normoxia.

**Fig 8:**
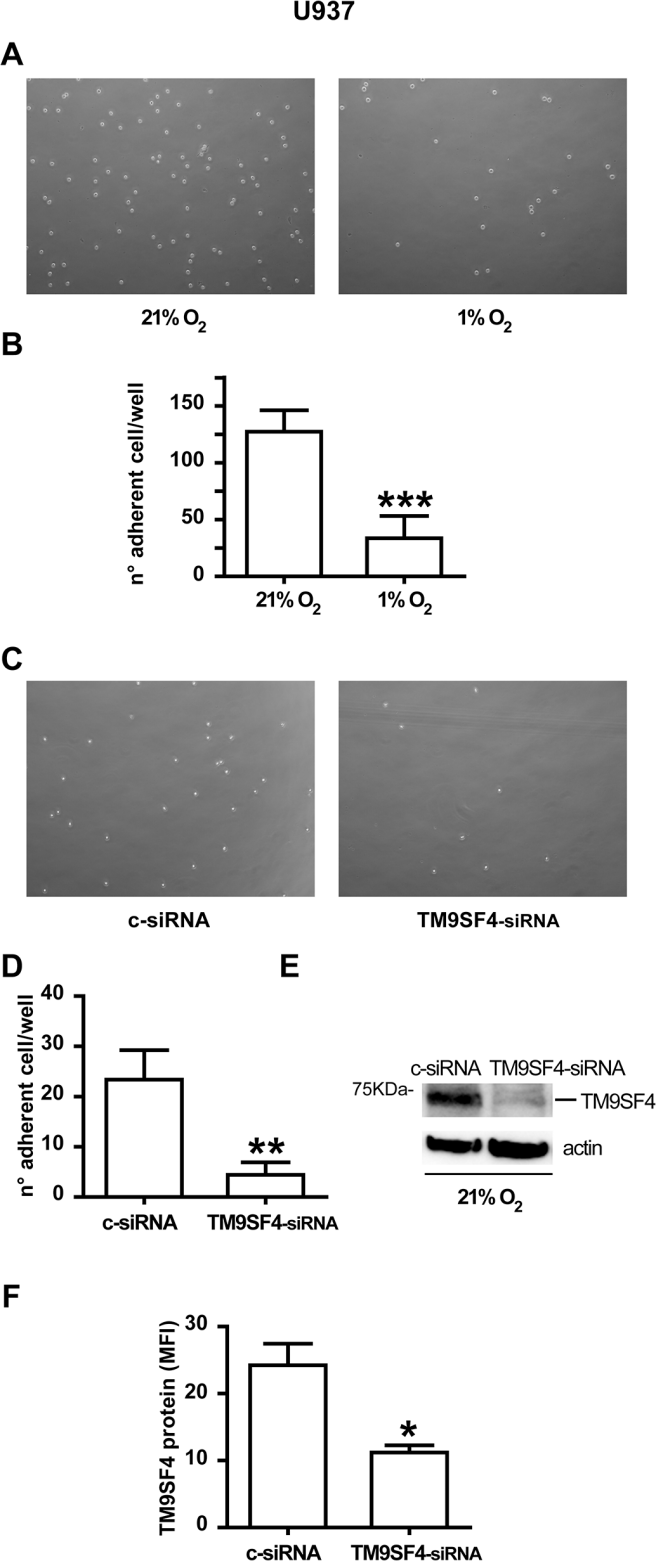
TM9SF4 is a new molecule involved in cell adhesion of leukemic cells. (A, B) Adhesion assays show that the number of adherent U937 cells to fibronectin (Fn)-coated plates under hypoxic condition (1% O_2_) is significantly decreased compared to normoxic conditions (21% O_2_). (C, D) In normoxia, adhesion assays show that the number of adherent TM9SF4-siRNAs transfected U937 cells to fibronectin (Fn)-coated plates (TM9SF4-siRNA) is significantly lower than the number of adherent control-siRNA transfected U937 cells (c-siRNA). (E; F) Knockdown of TM9SF4 expression in TM9SF4-siRNA transfected U937 cells (TM9SF4-siRNA) was controlled by Western blot (E) and flow cytometry (F) analysis of TM9SF4 protein expression compared to control-siRNAs transfected U937 cells (c-siRNA). (A, C) Pictures of one representative experiment of adhesion assays are shown. (B, D, F) Data are presented as the mean of six independent experiments (n = 6) ± SEM; **, *** are p<0.01, p<0.001 respectively. (E) One representative experiment out of three is shown; actin is shown as internal control.

Our data showed that in hypoxia the number of adherent U937 cells to Fn-coated wells decreases, as compared to normoxia (Fig 8A and 8B). Overall, we found that hypoxia induces a loss of cell adhesion ability (around 70%) to Fn of leukemic U937 cells, compared to normoxic U937 cells (Fig 8B).

To demonstrate that TM9SF4 is directly involved in cell adhesion to Fn, we performed RNAi (interference)-mediated TM9SF4 gene silencing in U937 cells and adhesion assays.

U937 cells were transfected with specific TM9SF4 interfering small RNAs (TM9SF4-siRNA), or with a non-targeting control siRNA (c-siRNA), and cultured on Fn-coated plates in normoxic condition (21% O_2_) (Fig 8C and 8D). Adherent cells on Fn were counted in both U937(TM9SF4-siRNA) and U937(c-siRNA) cell cultures (Fig 8D), while knockdown of TM9SF4 in U937(TM9SF4-siRNA) cells was controlled by Western blot and flow cytometry analysis of TM9SF4 protein expression, as compared to U937(c-siRNA) control cells (Fig 8E and 8F).

Our data showed that U937(TM9SF4-siRNA) cells have a lower cell adhesion ability on Fn than U937(c-siRNA) cells (Fig 8C), as shown by the count of adherent cells to Fn of these cultures indicating around 80% of inhibition of adhesion of U937(TM9SF4-siRNA) cells, as compared to U937(c-siRNA) control cells (Fig 8D).

Altogether our data indicate a role for TM9SF4 in cell adhesion of leukemic cells.

## Discussion

In human TM9SF4 was identified by its homology of sequence with phg1a, a gene essential for Dictyostelium adhesion and phagocytosis. Early studies on TM9SF4 expression and function performed in human malignant melanoma cells, have shown that TM9SF4 is overexpressed in metastatic cells, while undetectable in primary melanoma cells or healthy tissues, and is involved in an atypical phagocytic activity of these malignant tumor cells, called cannibalism [11–13]. Cannibalism that occurs when a tumor cell engulfs another cell [13] is supposed to be a crucial survival option for tumors in conditions of low nutrient supply and low oxygen tension or hypoxia, an important component of cancer microenvironment [14, 15]. Whether cannibalism is an exclusive property of human malignant tumor cells [13], autophagy could be assimilated to cannibalism in normal human cells [47]. A recent study provided evidence that TM9SF4 is a V-ATPase-associated protein that modulates drug resistance and invasiveness of tumor cells [16].

Hypoxia occurs in solid tumors and is not generally present in most healthy normal tissues. However, the BM where oxygen supply is limited, represents a somewhat unique tissue type where hypoxia is considered as a physiological condition, given its hierarchical organization emanating from a small population of resting hematopoietic stem cells localized in hypoxic niches [18]. BM microenvironment plays also a pivotal role in the initiation and propagation of leukemia. In fact, leukemic cells can infiltrate this microenvironment (niche) and may hijack the homeostatic mechanisms of normal hematopoiesis, leading to enhanced self-renewal and proliferation, quiescence, and resistance to chemotherapeutic agents. Then, the impact of hypoxia is currently under investigation in hematologic malignancies, especially in acute myeloid leukemia (AMLs) [26].

As in malignant tumor cells, TM9SF4 mRNA was found overexpressed in a small subset of AMLs and myelodysplastic syndromes (MDS) characterized by the amplification of a chromosome 20 fragment (20q11.21) bearing the entire TM9SF4 gene [17]. Although no data are available on TM9SF4 expression and function in normal and leukemic hematopoietic cells, these data suggested a potential role for TM9SF4 in leukemogenesis.

In our study, we reported for the first time that TM9SF4 is a gene expressed in human hematopoietic progenitor cells (HPCs) and regulated in a lineage dependent way during Mo and G proliferation and differentiation of HPCs.

Interestingly, we found that TM9SF4 expression is particularly high in CD34^+^ HPCs. TM9SF4 expression decreases during proliferation and selective Mo and G differentiation of CD34^+^ HPCs, thus suggesting a putative role for TM9SF4 in these lineages both involved in adhesion, immune function, and inflammatory response. However, during the late stages of granulocytic differentiation TM9SF4 expression is restored. In line with this last observation, induction of terminal granulocytic maturation of HL-60 cells by ATRA treatment is associated with a marked upmodulation of TM9SF4 expression.

By analyzing first TM9SF4 expression in a small panel of AMLs pertaining from the M0 to M5 FAB subtypes, we found that TM9SF4 is overexpressed in some AMLs, particularly in acute myelomonocytic M4 AMLs and also in M2 and M3 AMLs, as compared to normal CD34^+^ HPCs, thus indicating that TM9SF4 expression is deregulated in AMLs with a granulocytic component and could play a role in the development of these leukemias. Particularly, in line with a recent study [16], it is tempting to suggest a possible role for TM9SF4 overexpression in AML chemoresistance. Then, we showed that TM9SF4, clearly expressed in Mo and G leukemic cell lines, decreases during vit.D3 induced-Mo differentiation, while increases during ATRA-induced G differentiation of these cells.

By analysing the impact of hypoxia on TM9SF4 expression in leukemic cells, we found that TM9SF4 is downregulated in both Mo and G leukemic cells cultured in hypoxia, as compared to normoxia. Then, by analysing a partial promoter of TM9SF4 in which we found a HRE site, we identified TM9SF4 as a new direct target gene of HIF-1α in leukemic cells.

Since hypoxia-mediated downregulation of TM9SF4 in leukemic cells was associated with a decrease of adhesion on fibronectin, selective TM9SF4 knockdown mediated by RNA interference was performed, supporting a possible role of TM9SF4 in mediating adherence to fibronectin. These findings indicate that TM9SF4 could act as a new adhesion molecule. In line with our data, the function in adhesion and phagocytosis was first described for the evolutionarily conserved protein Phg1A, homolog of TM9SF4 in Dictyostelium [4, 6] and subsequently also observed in Drosophila haemocytes, immunosurveillance cells acting in a similar manner to cells of the mammalian monocyte and macrophage lineage [8], and later in human tumor cells [11]. Furthermore, it is of interest to note that Drosophila TM9SF4 mutant larvae failed to correctly encapsulate parasitoid wasp eggs [8], a process that requires Rac2 GTPase and integrin-mediated adhesion of other immunosurveillance cells called plasmatocytes to the foreign parasite [48, 49]. TM9SF4 was also considered a candidate for coupling changes in the actin cytoskeleton to adhesion and, putatively, for controlling integrin-dependent activation of Rho GTPases during the adhesion of plasmatocytes to pathogens [48, 50].

Altogether, these findings indicate a potential role for human TM9SF4 in integrin-mediated cell adhesion process, to further investigate [51].

In this study, we identify TM9SF4 as a new HIF-1α-responsive molecule, expressed in human hematopoiesis, regulated during Mo and G proliferation and differentiation of HPCs, and particularly overexpressed in AMLs with granulocytic differentiation capacities. TM9SF4 may be hypothetically considered as a potential oncogene, a function to be further investigated in leukemic cells.

## Supporting Information

S1 Fig Hypoxia and cell differentiation, two mechanisms to control TM9SF4 expression in leukemic cells. (A) Flow cytometry analysis of specific Mo markers CD11b and CD14 expression was performed from day 0 to 5 (d5) in U937 cells grown and treated with vitamin D3 (VITD3) to induce Mo differentiation under hypoxic conditions, as compared to untreated U937 control cells grown in hypoxia. (B) Flow cytometry analysis of G markers CD11b and CD54 was performed from day 0 to 5 (d5) in HL-60 treated with all trans retinoic acid (ATRA) for G differentiation performed in hypoxia, as compared to untreated HL-60 control cells grown in hypoxia (d0). (C) Western blot analysis of HIF-1α nuclear protein expression in U937 untreated (day 0) and VIT.D3-treated (day 5) U937 cells in hypoxia (1% O_2_). (D) Western blot analysis of HIF-1α nuclear protein expression in HL-60 untreated (day 0) and ATRA-treated (day 5) HL-60 cells in hypoxia. (E) Western blot analysis of TM9SF4 protein expression in U937 cells and during their VIT.D3-induced differentiation performed in hypoxia. (F, G) Western blot analysis of TM9SF4 protein expression in HL-60 cells (F) and HL-60(HIF-1α-siRNA) cells (G), and during their ATRA-induced differentiation performed in hypoxia. (A, B) The results of three independent experiments (mean ± SEM values) are shown; *, **, *** are p<0.05, p<0.01, p<0,001 respectively. (C-G) One representative experiment out of three is shown; (C, D) nucleolin is used as an internal control of U937 and HL-60 nuclear protein extracts; (E-G) actin is shown as internal control of total protein extracts.
(TIF)
