# Supplementary figures and images for: Human TM9SF4 Is a New Gene Down-Regulated by Hypoxia and Involved in Cell Adhesion of Leukemic Cells
Source: PLoS One. 2015 May 11;10(5):e0126968. doi: 10.1371/journal.pone.0126968 (PMC4427288; doi:10.1371/journal.pone.0126968)

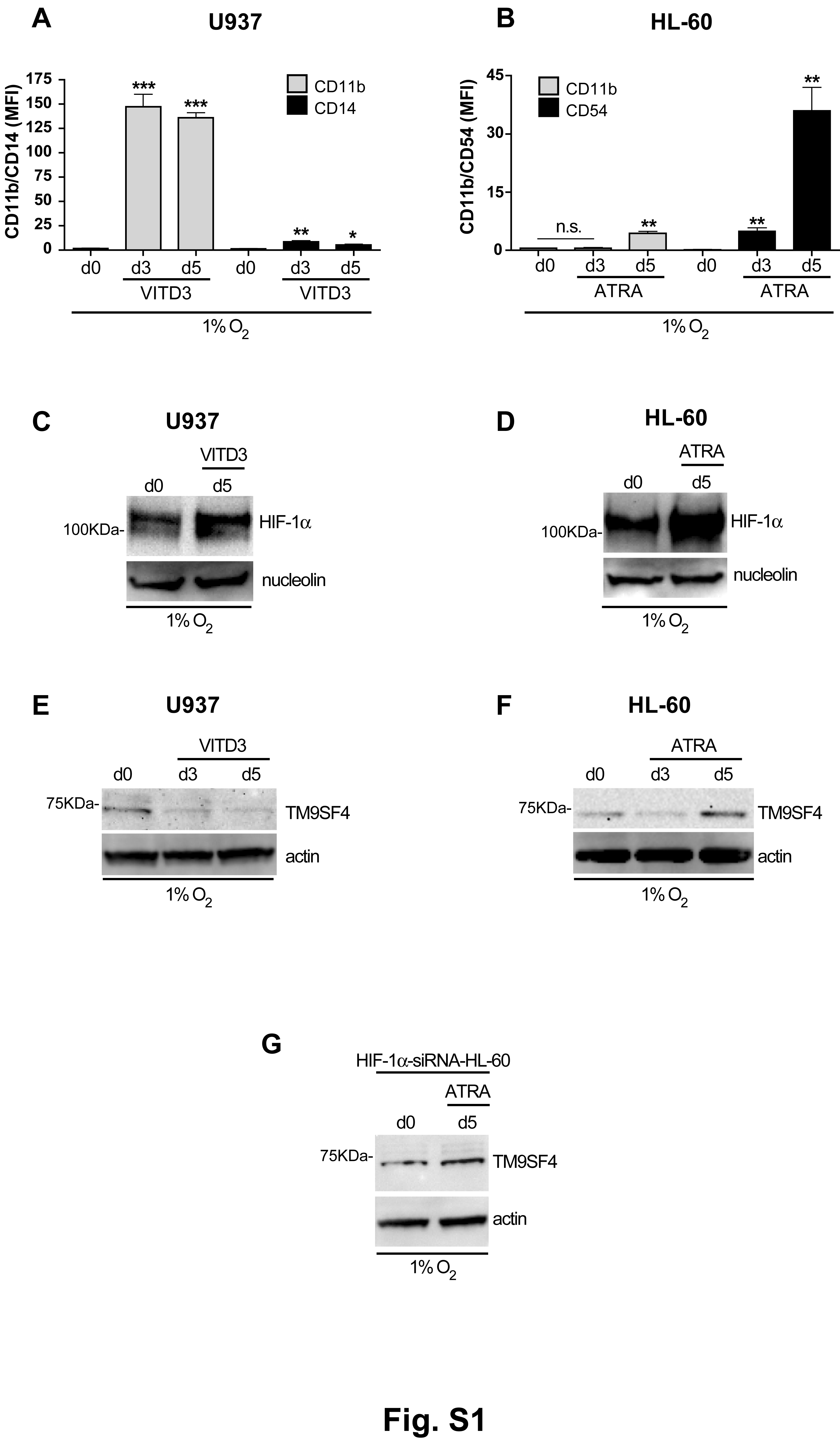

Supplement: S1 Fig — (A) Flow cytometry analysis of specific Mo markers CD11b and CD14 expression was performed from day 0 to 5 (d5) in U937 cells grown and treated with vitamin D3 (VITD3) to induce Mo differentiation under hypoxic conditions, as compared to untreated U937 control cells grown in hypoxia. (B) Flow cytometry analysis of G markers CD11b and CD54 was performed from day 0 to 5 (d5) in HL-60 treated with all trans retinoic acid (ATRA) for G differentiation performed in hypoxia, as compared to untreated HL-60 control cells grown in hypoxia (d0). (C) Western blot analysis of HIF-1α nuclear protein expression in U937 untreated (day 0) and VIT.D3-treated (day 5) U937 cells in hypoxia (1% O2). (D) Western blot analysis of HIF-1α nuclear protein expression in HL-60 untreated (day 0) and ATRA-treated (day 5) HL-60 cells in hypoxia. (E) Western blot analysis of TM9SF4 protein expression in U937 cells and during their VIT.D3-induced differentiation performed in hypoxia. (F, G) Western blot analysis of TM9SF4 protein expression in HL-60 cells (F) and HL-60(HIF-1α-siRNA) cells (G), and during their ATRA-induced differentiation performed in hypoxia. (A, B) The results of three independent experiments (mean ± SEM values) are shown; *, **, *** are p<0.05, p<0.01, p<0,001 respectively. (C-G) One representative experiment out of three is shown; (C, D) nucleolin is used as an internal control of U937 and HL-60 nuclear protein extracts; (E-G) actin is shown as internal control of total protein extracts. (TIF) [file pone.0126968.s001.tif]
